# On the Same Wavelength? Differing Geopolitical Positionalities and Voluntary Return and Reintegration in Ghana

**DOI:** 10.1007/s12134-022-00958-x

**Published:** 2022-04-26

**Authors:** Ester Serra-Mingot, Markus Rudolf

**Affiliations:** 1 Faculty of Sociology, Bielefeld University, Postfach 10 01 31, 33501 Bielefeld, Germany; 2 Bonn International Centre for Conversion, North Rhine-Westphalia, 53121 Bonn, Germany

**Keywords:** Return migration, Reintegration, Ghana, Assisted voluntary return and reintegration (AVRR)

## Abstract

This paper explores the range of experiences of “voluntary” return to Ghana, based on the different positionalities of migrants set against migration and return regimes and broader socio-economic inequalities. The cases illustrate how geopolitical relations shape migrants’ mobilities, highlighting the unequal relations between different actors in the countries of origin and destination; primarily these are the migrants, their relatives, and communities of origin. Conflicting interests and expectations of these actors, as much as differing return policies, create unequal options and expectations of mobility. Migrant journeys, regardless the age, gender, legal status or social class, are always geopolitical journeys. The diverging experiences of return, thus, depend not only on the individual situations, but also on the broader politicized relations and interests between stakeholders in the migration and return processes.

## Introduction

To commemorate the 400th anniversary of the arrival of African slaves in America, in September 2018, Ghana’s President, Nana Akufo-Addo, declared 2019 as the Year of Return. It was intended to encourage people of African descent (mostly African-Americans) to return to their home and reclaim their identity (Yeboah, 2019). It was evident, however, that the initiative also had financial implications, in that it encouraged people in the diaspora to settle and invest in Ghana and contribute to the country’s development (Rabaka, 2020). The year-long campaign, which included a full calendar of events (e.g. art exhibits, visits to heritage sites, creative economy and trade conferences) was considered a great success by the Ghanaian government. According to the Ghana Immigration Service, the total numbers of visitors from the USA (26% of the visitors) the UK (24%), Germany (22%), and Liberia (14%) grew considerably (Yeboah, 2019) from levels recorded in preceding years.

In the same year, the German Federal Ministry for Economic Cooperation and Development (BMZ) commissioned the “Migration and Diaspora Programme” to harness the positive impact of regular migration and diaspora engagement to drive forward social and economic development in partner countries (Ghana being one of them).^1^ This programme was the successor of the previous one "Migration for Development"^2^ (2017–2020), which was part of BMZ’s “Returning to New Opportunities” programme. It aimed at improving the prospects for economic and social participation in selected countries of origin for the local population and returning migrants (with focus on those in an irregular situation). These programmes reflect the goal of the German Government’s migration policy to leverage the economic potential of regular migration to strengthen partners’ governance capacity to shape migration processes, to address the causes of irregular migration and to support migrants who are trying to return and reintegrate into the labour market (Biehler et al., 2021).

Whereas the main goal of the campaigns of the governments of both host and origin countries is to foster return migration to encourage some kind of development,^3^ domestic political discourses around these issues widely differ. Perceptions of migration, return and reintegration among the countries of origin and destination furthermore vary between and within the destination countries according to the social, political and economic position of the migrants and their relations with the respective receiving communities. The particular developmental impact of migration and return for the larger society is seen differently by migrants and returnees, hosting communities, and various non-governmental organizations in the countries of origin and respectively of return.

Against this backdrop, the paper examines the challenges of “voluntary” returnees in Ghana that result from these often conflicting individual and institutional geopolitical positionalities with regard to “voluntary” return. Based on qualitative fieldwork conducted with Ghanaian voluntary returnees (with and without the support of Assisted Voluntary Return and Reintegration [AVRR] programmes) from the USA, Libya and Germany, this paper shows how a lack of consensus, as much as differing interests and expectations of the actors involved, undermines different reintegration initiatives. The article contributes to the current debates on return and reintegration by delivering empirical examples of, firstly, the contrasting narratives prevailing in the Global North and South, secondly by setting the resulting approaches and policies in relation to the de-facto impact of AVRR, and thirdly by assessing the outcomes of reintegration initiatives from a bottom-up perspective.

After pointing at the benefits of applying a geopolitical perspective on migration and return and shortly laying out our methodology, we discuss the everyday experiences of return referring to three individual cases that set the stages for analysing the relation of return, development of the place of origin and the importance of circular movements. Our main argument is that migrant narratives illustrate how relations and negotiations between states influence every step of the migration process thereby manifesting the role of geopolitics in their trajectories. The expectations of migrants, returnees, and stayees are embedded and conditioned by the wider context of perspectives on migration and return that prevail in the respective societies. They differ between the countries of origin and destination in regard to the social, political and economic position of the persons transcending national borders and the relations with respective receiving communities.

## Migration and Return as Multi-stakeholder Geopolitical Processes

In the Global North and Middle East, labour migrants and refugees from the Global South, including Ghana, are typically categorized as mostly unskilled and cheap labour needed for construction, agriculture, and the domestic service sector (especially in the Middle East) – or as criminals. In the EU, a particularly prevalent narrative circles around concerns regarding the cost that migrants cause for the social welfare system (Borjas, 1999; Razin & Wahba, 2015). Hans Lucht, for instance, has shown how Ghanaian migrants get trapped in circumstances where such notions have effectively halted individual aspirations and created frustrations. The fact that virtually only low-skilled jobs at the fringes of the host societies (e.g., the underground informal economy in the case of his ethnographic research in the South of Italy) are available lead to unmet expectations (both of the migrants and their families) and rising frustrations also on both sides (Lucht, 2012).

From a Global South perspective, migration—regular or not—is often a viable financial strategy for development. Both individual households and governments benefit directly from the transfers of goods, ideas and resources (Mouthaan, 2019). In Ghana, the communities abroad are mostly regarded as income providers (Bob-Milliar, 2009; Bob-Milliar & Bob-Milliar, 2013a; Kandilige & Adiku, 2019). Another difference is related to citizenship, nationality and residence. Citizenship in the Global North refers mostly to a community of loyal taxpayers entitled to state services through residence, nationality or both. In Ghana, in contrast, the community of citizens is foremost defined by direct or assumed kinship—sometimes, as in the case of the diaspora, regardless of residence or nationality.

When it comes to return and reintegration, migration policies in the Global North have become more and more influenced by domestic politics. In the last five years a more-or-less global tendency has emerged that aims at lowering the number of irregular migrants staying or coming into the NAFTA or EU. For politicians and policy makers in the Global North, sustainable return is often synonymous with the returnee not re-migrating (ICMPD, 2015; Kuschminder, 2017). Successful reintegration, from this point of view, is achieved when irregular migrants leave the chosen destination to return to the place of origin and stay there permanently. The EU, for example, defines sustainable return as “the *absence of migration after return* [emphasis by the author] because the returnee is fully integrated socially and economically in the home community” (Publications Office of the European Union, 2013, 7). According to this logic, re-emigration, becomes an indicator for unsustainable return processes (European Migration Network, 2016).

This is however slowly changing. Re-migration, according to IOM's 2017 definition of sustainable return and reintegration, only represents a “failure” if it occurs through necessity rather than choice. Scholars have likewise shown that, for return to be sustainable, returnees need to retain access to the wider international professional and social network in the places where they have worked and lived (Black & King, 2004). This argument resonates with Ghanaian concepts on return where returnees are typically seen as investors bringing in skills, resources and innovations. The national Ghanaian migration policy, thus, encourages the return of the diaspora in order to contribute to national development (Kandilige & Adiku, 2019; Kleist, 2015) and political leaders are increasingly held accountable by the constituencies if they fail to advocate in the interest of their fellow-country people abroad.^4^ Ghana’s interest to support exclusionary and permanent return measures or deportations by OECD countries is therefore often limited.

In regard to migration and return policies, in sum, two-dimensional perspectives prevail. In the Global North spatial (moving outside space A) and time criteria are combined negatively (remaining outside A) to define sustainable migration regimes. Social, economic, and psychological aspects are side-lined and only come into the equation when causing re-migration (to A). In the Global South, assessments of migration are at first glance the exact opposite of those from the destination countries. Yet also in the countries of origin a bipolar model prevails on a policy level, in which migration and return are evaluated by their economic benefits or costs, disregarding the non-economic causes and impacts of migration. The idea to migrate or to return is not static, as numerous academics have shown.^5^ Nowadays, return migration is seen as a stage in ongoing cycles of mobility rather than a final end-point of a previous migration (Kleist, 2018).

## Methodology

The findings of this paper are based on qualitative fieldwork conducted in Ghana from September 2019 to June 2020. In-depth qualitative interviews, informal conversations and observations were conducted with 35 Ghanaian returnees that auto-labelled their return as voluntary (11 of whom were beneficiaries of AVRR support, 18 women, and 17 men. The age ranged from persons in the twenties to their sixties, and the sample covered 3 that went to neighbouring countries, 3 that headed to the middle East, 4 to North America and the rest—including multiple transit countries—that had been to Europe), as well as with members of the local communities in Greater Accra, Ashanti Region and Central Region. The interviews were conducted by the two authors together with two Ghanaian assistants in English and Twi. Respondents were selected through purposive sampling and systematic serendipity. For those with AVRR support, access to respondents was facilitated by international organizations, such as the IOM and the Ghanaian German Centre, while those without were found with the help of informal contacts, local community organizations and through the snowball method.^6^

Our participative research provides representative, ideal–typical cases without claiming statistical representativeness. The study tried to adhere to the principles of approaching the field in the most unbiased and reflective manner as possible. We paid attention to differences of ethnicity, gender, age and diversity, guaranteed the anonymity of all respondents, tried to establish an atmosphere of trust and sought an open dialogue. All respondents were informed orally and on paper about the study’s content, objectives, conditions of confidentiality, and the opportunities to withdraw their consent at any time. The cases illustrate the interactions of Ghanaian returnees with the current structures of return and reintegration. Returnees came from three main destination countries, Germany, Libya^7^ and the USA, which have been traditional destinations for Ghanaians of all class and age ranges.

To understand how returnees navigate the geopolitical context of migration governance, we borrowed from feminist geopolitical scholarship, which stresses the relevance of understanding the “everyday and embodied sites and discourses through which transnational economic and political relations are forged and contested” (Williams & Massaro, 2013: 753). This approach allowed us to address both the reproduction of geopolitical relations and the individual’s resistance to them (Ashutosh & Mountz, 2012). Feminist scholars argue that traditional analyses of geopolitical relations fail to address the lived realities of individuals and communities, offering only a “disembodied critical practice” (Williams & Massaro, 2013: 753). Looking at returnees’ experiences is, thus, relevant to address both the reproduction of geopolitical relations and the resistance to them. It is furthermore essential to connect the political representation to “geographies of everyday life” and to look at the manner “in which the nation and the international are reproduced in the mundane practices we take for granted” (Dowler & Sharp, 2001: 171). This perspective allows us to scrutinise the “uneven ways in which geopolitical processes shape the lives of differently situated populations, drawing attention to how even the most intimate and everyday aspects of life are key sites where geopolitical power is (re)produced and negotiated” (Williams & Massaro, 2013: 753).

## Setting the Context: Migration, Return, and Reintegration in Ghana

Ghana has a long tradition of both voluntary and forced outward migration, at a national, regional and international level (Akyeampong, 2000). Whereas historically, most migration flows from Ghana were regional due to commerce, forced labour and circular nomadic routes (Grillo & Mazzucato, 2008), over recent decades migration patterns have extended geographically. Nowadays, out of 31 million Ghanaians, around 1.5 million are living outside Ghana, of whom about 70 per cent live in the Economic Community of West African States (ECOWAS) zone, followed by OECD countries in a much smaller scale (Mouthaan, 2019). Migration thus emerged as a strategy for dealing with economic and social challenges back home.

In the early 1980s, political oppression and widespread poverty, coupled with the expulsion of almost two million Ghanaians from Nigeria, led large groups of educated and politically engaged Ghanaians to leave the country and seek asylum, mostly in Germany, the Netherlands, the UK and the US (Schans et al., 2013). Facing the economic crisis and widespread unemployment in Ghana, since the 1990s and up to today, a large number of primarily young Ghanaian men have migrated to Libya in search of better job opportunities (Bredeloup & Pliez, 2011; Hamood, 2006; Kleist, 2018). In some cases, after having worked for some time to save money for the onward journey, Ghanaian Libya-bound migrants try to reach Europe across the Mediterranean while others return repeatedly (Bob-Milliar, 2012; Mensah, 2016). Despite the ongoing conflict and associated human rights’ violations, this practice could still be observed in our field research in 2020. The different destinations and ways of migrating reflect the unequal access to safe and legal migration between Western countries and Africa, which at the same time result in different migration projects and trajectories of return (Kleist, 2018). What can also be implied is that Ghanaian migrants are far from being a heterogeneous group—there are highly skilled persons, students, traders and asylum-seekers next to low-skilled labour migrants (Kleist, 2018).

Migration is, nevertheless, no one-way-street: remittances, investments, visits, circular migration and return to Ghana have considerable social and economic impacts (Hall, 2018). In fact, reasons to return are as diverse as reasons to migrate, and so is the motivation to remain in Ghana or plan further movements. Factors such as gender, age, social class, educational level, the existence and geographies of supportive social networks, the legal status during migration and after return, and the availability of state-based return schemes are some of the factors affecting the ways in which return and reintegration are experienced.

Another important point to take into consideration is the question of “voluntariness” of return that these projects involve. In fact, despite technically being an important part of AVRR programmes, the extent of “voluntariness” and “choice” of returnees has been questioned (Salihi, 2021, Biehler et. al. 2019, 2021). Concluding that AVR returns are hardly voluntary at all, some authors have suggested to only speak of assisted return (Kuschminder, 2017: 14). Some of the cases we present here confirm that the label voluntariness is more often imposed than inquired. In other words; Return was not experienced as voluntary if the process lacked a certain degree of (mental and/or physical) preparedness and if the individuals felt that no alternatives existed. is It is due to these observations on the degree of free and unconstrained choice to return was for many not existent that we use the word “voluntary” with inverted commas.

Studies show that returning empty handed caused family rejection or other reintegration problems and put returnees in a highly vulnerable position (Hall, 2018). Our results show that despite such returns being seen as disgraceful by both the returnees as their social contacts, many return migrants were able to count on the support from family members, friends, colleagues, and social organizations and networks. Our observation clearly indicated that this support is often directly related to remittances or migrants’ direct investments that fostered the development of the community into which they reintegrated. Vice versa, recent studies have shown that those who had “failed” abroad were usually considered more of a burden for their families (Hall, 2018). We observed this burden to directly correspond to the economic loss caused by the “failure.” If, on the contrary, economic costs were in balance, respondents reported that their families were happy to see them safe back home again. The warm welcome was reported even if all of the remittances meant for investments had been “eaten” and even though the return meant a loss of future remittances for the welcoming communities. This shows the interrelatedness of social and psychological dimensions of reintegration with economic aspects that, in turn, stretch back to the beginning of the migration process.

## Everyday Experiences of Return and Reintegration

In order to analyze in depth how returnees navigate the geopolitical context of migration governance, it is crucial to assess “… everyday and embodied sites and discourses through which transnational economic and political relations are forged and contested” (Williams & Massaro, 2013: 753). In the following sub-sections we, thus, scrutinize the geopolitical processes that frame the everyday experience of migrants and their positionalities, and concretize where power is produced, reified, and negotiated. The three presented cases were chosen to illustrate a bandwidth of trajectories stretching from officially recognized and supported returnees to those moving under the radar. The results in general confirm that involuntary return disrupts, slows and hampers migration projects but does not necessarily end them (Kleist, 2017, 2018; Mouthaan, 2019). They cases furthermore corroborate findings showing that return caused conflict and economic strain, especially for female family members in poor families where remittances play a central part (Kandilige & Adiku, 2019; Mensah, 2016). As described in the literature we found migration to be a means to become a proper and respectable adult man – manifested in extensive social relations that in turn reify hegemonic masculinity ideals. This relates to a wider ranging argument from studies on highly-skilled Ghanaian returnees from the Global North about return processes involving renegotiations of gender identities, roles and norms (Kandilige & Adiku, 2019; Mensah, 2016).

### Returning to Save One’s Life—Juliet

After spending four years in Germany, Juliet (59) returned to Accra in September 2019 as part of an AVRR programme.^8^ Juliet had secondary education, and by the time she left for Germany in 2015 she was a widow and a mother of three children, all of them over 18. Juliet decided to migrate when her niece, who had married an elderly and well-off German man, asked her to come to take care of her husband. He took care of Juliet’s tourist visa and flight to Germany. When Juliet’s tourist visa expired, she remained in Germany as an undocumented person. As she was a full time in-house care worker, this was not problematic for some time. However, in 2017 Juliet fell out with her niece, and she was asked to leave the house immediately. Undocumented, by then sick, and not knowing what to do, Juliet spent time on the streets or at friends’ homes. As other returnees explained, hosting undocumented people in Germany is penalized, so they can only be hosted by friends and acquaintances for very limited periods of time, and often by accepting exploitative arrangements. For instance, several respondents reported being charged exorbitant rents, and especially women talked about being pushed to carry out sexual favours. At some point Juliet was offered to marry a Ghanaian-German man in order to get her papers, but she refused.

It was then that she asked the pastor of her church in Germany for advice. Given her delicate health situation, the pastor advised her to ask for asylum in order to be, at least, taken care of. After her claim for asylum was rejected, she was offered the possibility to return to Ghana as part of the AVRR programme, which she accepted. This situation was experienced by most of our respondents who had benefitted from AVRR. Some of them also explained that the sooner one *accepts the deal,* the more support one can receive. In 2019, once Juliet’s health situation had stabilized, she returned to Ghana. The German government, through the IOM’s AVRR programme, paid for her flight and she received 300€ to buy clothes and other necessary things for the trip. She also got a document which entitled her to receive up to 2,500€ in-kind support to set up her business upon arrival. Before boarding the plane in Germany, the IOM gave her an additional lump sum of 1,000€ in cash.

By the time we interviewed Juliet in Accra, in February 2020, she was still waiting for the in-kind support to start her business. Whereas she felt grateful for the cash she had received, she lamented that this money had not been enough to also support other relatives or the broader community, whereby she had experienced a certain grade of exclusion: “Everyone rejects you if you don’t bring money. They don’t mind me, they don’t accept you. They think you’re useless because you bring nothing.” For the time being, although Juliet was living for free with one of her children, the 1,000€ she had been given were coming to an end, mostly because a lot of the money had gone to pay for her medicine. By July 2020, Juliet had finally received the full amount of the in-cash and in-kind support. However, by that time, due to Covid-19, Juliet’s business of providing school material had not yet been able to properly start.

### Returning for the Family: Amadou

As mentioned, sudden and unplanned returns—as after the Arab spring in Libya—often cause a considerable loss of respect among close relatives for returnees. This is mostly due to the fact that the remittances had considerably improved the life of extended families. The very nature of the circumstances of return nevertheless also play a significant role—even though many migrants on the one hand reported to be only seen as a source of remittances, many family members on the other hand insisted that they are mostly worried about the wellbeing of their kin. Amadou (33) was born in the northern part of Ashanti Kwahu region and has two children that live with their mother in Accra. Growing up with his grandmother after he lost his mother at the age of three, he stopped attending school at junior secondary school to help his grandmother who was selling charcoal. After some years he joined the truck driver that used to collect the charcoal from his grandmother’s place and arrived in the Accra neighbourhood frequented by Ghanaians from the northern part of the country. Working his way up in the system of referrals and references as a freelancer, he became an auto-mechanic and specialized in electrics for trucks and coaches. He used to move up and down the entire territory of Ghana as a mobile mechanic fixing buses when they broke down on the road.

At the interview in January 2020 in Accra, he recounted that he was sought after in this profession. When the first private transport line in Ghana was set up to import VIP buses from China, the company wanted to hire him. Yet he missed this opportunity because he did not have a passport to go to China and check the air conditioning and heating systems for the company. He explained that he then went for a passport, in order “not to miss this chance again” and elaborated that “this is when the idea of travelling occurred to me.” Seeking advice, a driver who had been to Libya recommended him to go to this country and helped him arrange the journey. When embarking on this trip, he nevertheless got lured into a trap and was held hostage upon arrival in Libya before his family managed to pay for his ransom through middlemen. After surviving the agony of incarceration he made it to Tripoli where he worked as a mechanic and in the construction sector for two years. Because of the overthrow of Gadhafi, his family urged him to come back. Upon return he found out that he had no savings:

I had been sending money home to my brother who instead of saving had put it into a business that crashed. So when I informed him that I would be coming home, he welcomed the idea but told me that my money is gone. I had sent roughly 3,000 Cedis (440€ approx.) home. My brother had contributed to the upbringing of my children. The salary came out of the construction work. There is a measure [of the accomplished work] by meter. We work in a group and you share after you finish the work. You get between 1,000 (145€) and 1500 Cedis (220€) for six months. We sleep at the site and we are brought food. As a mechanic I got 800 Cedis (110€) for a month and slept at the company site. The company drained the water from the desert and I did the boreholes. It was a good company.

Amadou says that he still desires to travel, to explore and to find “a better platform to demonstrate and use my capacities.” He explains that a friend went over the sea to Germany and is now doing well there. When the friend came back to his home town, he set up a company with electronics. Amadou explained that many others from his village went to Europe as well. When they come home for holidays, they build two-bedroom apartments. According to Amadou, this motivates him to go to Europe as well. Since he returned, he nevertheless managed to establish a livelihood. He imports cars from the USA, then repairs and sells them for a commission. His family, he explains, “…were happy to have me back, still alive, even though I didn’t bring anything”. After commuting between his home town and Accra for a while, he is now renting a single room. He had to pay for two years in advance – and he makes around 500 cedis (72€) a month plus extras. If things go well he plans to marry the mother of his children who is currently staying with her mother.

### Returning to Develop: Rob

Rob (39) was born in Germany while his parents worked and studied in this country for many years. When Rob was two years old, the whole family returned to Ghana. It was in 2004 that Rob, encouraged and supported by his parents, moved to the USA to take his Bachelor degree. Upon completing it, he continued to work in the USA, where he also had a daughter out of a now broken relationship. In 2007 his father died, leaving Rob and his brother in charge of the family car-towing business. Initially, Rob supported his brother, who was in Ghana at the time; Rob remained in the USA and only returned to Ghana in 2012 to work with his brother. Yet, they saw how all their efforts failed due to corruption. This did not allow them to further expand their business as planned, but also put their whole financial situation at risk. By the time we met Rob in February 2020 in Accra, his brother had given up and left for the UK. Rob, on his side was considering to leave Ghana as well:

So I want to transition there, get a place there, get a house there in the US, get everything settled, just like I’m living here, a car, a house so that when I moved there I can be relaxed and my mind can actually think what are my options, what kind of business I can set up, what can I do here. I think it’s possible to interact with both sides, I can be there, I can be here. I think that ultimately that’s what’s gonna happen. So I’ll be here maybe six months in the year and then move there maybe for three months, come back again.”

In fact, the “transition” between migration and return was something very important for Rob, as well as for many other interviewed Ghanaian returnees. This led to an evaluation of the general difficulties of re-migrating from USA to Ghana:

I always advise people that if you’re doing something there [abroad] and you want to move to Ghana, you must have a good reason to move here. If you have family, and you wanna bring your whole family here, then you must be very careful, because Ghana is expensive; to come and get a job here is not that easy as well. So if you’re coming to start something on your own, you must really plan it out well, ’cause if it fails, you have a lot of people depending on you. So I always tell people to study. Come down, spend some time, look around, look at the country, see what’s happening around, and get a feel for it. Spend some time, not just a short time, spend some time, and then keep coming and going and then if you feel like it is something that you can actually hop into, then you do it, but I can’t really advise you, yes, Ghana is doing so well, jump on a plane and come! No, because most of the times it doesn’t work, people come and left at two years. So, I will tell you that 90 per cent of my friends that moved back to Ghana have moved back [to the USA].

## The Geopolitics of Return and Reintegration

Like Rob, many highly skilled returnees in this study highlighted the desire to “develop Ghana” as one of the motivations to return. This fits the discourse of both the Ghanaian state (e.g. Year of Return) and the German state—as well as other EU states—for instance, through the Migration for Development program. But Rob’s experience shows that this wish is difficult to turn into reality. The cases described also help to identify other discrepancies between the policies designed for the integration of returnees and, respectively, their contribution to the development of their countries of origin and their everyday experiences.

### Developing Home

The debate on migration and development has been swinging from the developmentalist optimism of the 1950s and 1960s, to the pessimism of the 1970s and 1980s, and back again towards again more positive views in the 1990s and 2000s (de Haas, 2010). Nowadays governments of migrant-sending countries largely dismiss concerns on issues such as the so-called brain drain and focus on the potential of transnationally oriented migrants as actors of development instead. Through their remittance-sending practices, migrants are considered a more effective instrument for income redistribution, poverty reduction and economic growth than large, bureaucratic development programs or development aid (de Haas, 2010). Empirical evidence, ours and from other studies, however points to a more heterogeneous impact of migration on development (Agunias, 2006; Appleyard, 1992; Binford, 2003; de Haas, 2005). Recent views celebrating migration as an efficient form of self-help development from below should therefore be taken with a pinch of salt. Not the least because such views shift the attention from structural constraints that fall within the responsibility of states (de Haas, 2010; Kleist, 2008, 2017; Sinatti, 2015).

The debate on return migration and development mostly focuses on ways in which returnees can have a potentially positive impact on development. Our cases show that this, in practice, means returning with substantial savings and/or with newly acquired skills (cf. also Olivier-Mensah, 2019). Both of these potential benefits are influenced by the geography of return, which determines where, how and with whom the benefits will be enjoyed.^9^ The financial possibilities of the returnee and his or her dependents play a crucial role in this matter, as our cases show. These issues have been crucial ever since and had already been highlighted by a World Bank report from 2001 that found that Ghanaian returnees were more likely to establish themselves as small entrepreneurs if they returned with more than US$5,000 (Plaza et al., 2016).^10^

Another study, however, showed that relevant work experience was actually a more important determinant of entrepreneurial activity amongst Ghanaian returnees than education per se (Black & Castaldo, 2009). This study concentrated on voluntary returnees who were able to decide the time of their return and had opportunities to remain legal residents in the countries from which they returned. The relevance of skills is also clearly demonstrated in the three described cases. Rob’s case shows how highly skilled returnees are able to set a foot in the Ghanaian market – even though the benefit remains below expectations. Amadou’s case also demonstrates the role of skills: he managed to get back into his former field of activities. Juliet, however, faced uncertain prospects for an enterprise she had no experiences of as yet. Despite IOM’s support, thus, Juliet’s case shows that skills and counting with some sort of “financial mattress” when one goes back home are highly important to guarantee a smooth success. Skilled and unskilled returnees we interviewed were nevertheless united in lamenting about difficulties imposed by either the Ghanaian state (mostly due to corruption) or the German state (or whichever EU country they had migrated to).

### The Importance of Circular Movements Challenging the Idea of “Return”

Some independent studies of post-return on AVRR schemes propose a new framework for defining and measuring sustainable return, differentiating three dimensions: economic, socio-cultural, and safety/security (Koser & Kuschminder,^ 2015^). Koser and Kuschminder link the decision of a migrant to get involved in an AVRR programme with a lack of integration in a destination country. They highlight experiences of brain waste and de-skilling that reduce the chances of reintegration on return. The need to repay debts, the existence of new transnational ties and the social stigma attached to a perceived failed return have been identified as additional factors that make post-return reintegration more challenging (Collyer, 2018). The question, though, is whether a one-dimensional evaluation of return (A to B) is realistic. As for the long-term prospects of reintegration, there is also only very limited evidence on the conditions of returnees after return.^11^ Our research showed that virtually no returnee had returned to living the same life as before, cutting all strings attaching them to the experience abroad, or completely dismissed (re)migrating ever again.

According to our observations, returnees are rather interested in keeping doors open. The results show, as in the aforementioned definition of IOM, that it is actually the alternative of choice versus necessity that defines the success and the sustainability of return and reintegration. This is also confirmed in the literature. In their study on pre-and post-return experiences of Ghanaian international migrants, Setrana & Tonah (2016) looked at returnees’ assets and labour market participation, and found out that many keep ties with host countries for the sake of businesses and other benefits which may not be available in the home country. This is also confirmed in observations by recent studies (Bob-Milliar & Bob-Milliar, 2013b; Olivier-Mensah, 2019; Olivier-Mensah & Scholl-Schneider, 2016) that, like our own research, found returnees prefer to keep options for re-emigration open.

Like many undocumented migrants in Europe, such as Juliet, a common feature of lower class and unskilled migrants who return from African and Middle Eastern countries is that they are often able to raise fewer resources—if any—than their documented counterparts overseas, and they do not normally have the chance to regularly return home. This has multiple implications for the migrants and their families. On the one hand, several of our interviewees who had migrated to the Middle East or Libya, explained how, upon their return, they had found unexpected situations. As Amadou’s case illustrates, it was only upon his return from Libya that he realised his financial investment back home had been mishandled and lost. Other respondents in a similar situation to Amadou’s also reported non-financial “surprises” upon their return. In particular, two of our respondents, one returning from the Middle East by his own means, and the other one from Europe with the support of an AVRR programme, described how their their fiancées (sometimes with children) had “moved on” with another man during the time they had been abroad. This was both linked to the lack of remittances, but also to the lack of regular visits. On the other hand, the exploitative contracts many of these migrants have to accept (due to the lack of papers) do not allow them to send more than meagre amounts back home. Only very few manage to save enough to overcome the threshold to start a career, make an investment or start a business in Ghana.

## Conclusion

Applying a geopolitical perspective on the everyday experiences of migration and return, our study, on the one hand, showed the crucial role of international politics for the migration process, in particular the ever-negotiated relations between the involved states. The results, on the other hand, also indicate the role of local concepts on potentials, benefits and risks of migration and return that shape the strategies of the involved actors on the social, political and economic level. The respective concepts, in turn, differ between the countries of origin and destination and the respective sending and receiving communities, whose characteristics need to be integrated into any analysis as a third interrelated level. For the analysis of how these dimensions interrelate and how migrants deal in specific with the state—and other actors involved in the (return) migration infrastructure (e.g., IOM, Third Sector organizations)—feminist-geopolitical approaches point at the need to connect political representation and everyday life to contextualise matters: The presented narratives of the efforts of returnees to navigate the arena of migration management accordingly show a high capability to circumvent the presence of the state, but they also how states succeed in curtailing migrants’ mobility by denying or granting access to the national territory.

Our results show that the relations between geopolitics and migrant mobility are multifaceted. Migrant journeys, regardless of age, gender, legal status or social class, are always geopolitical journeys. As migrants navigate different geopolitical terrains, they are both authors of their own narratives, and expressions of broader geopolitical relations at work that structure their mobility (Ashutosh & Mountz, 2012). The cases of Juliet, Amadou and Rob show different ideas and experiences of return, which depend not only on individual situations, but also on the broader politicized relations and interests between national governments of both sending and receiving countries. The discussed migration trajectories are shaped by spatial impositions of power on mobility and the human agency of individuals and communities navigating geopolitical hierarchies. To assess the involved power relations, respectively the bandwidth of agency within it, both interrelated dimensions need to be taken into account (and related to the respective local social context – see below).

In other words: The presented cases illustrate the need to simultaneously dissect and relate the interdependent roles of skills, class, gender, geopolitics, legal status, and networks for return and reintegration: In regard to studies on highly skilled migrants that made it to the Global North, our findings confirm that unskilled migrants cannot easily capitalize on their class status, social and transnational networks. This corroborates doubts about the fluidity of social class in general, but the cases presented also show that it is, in exceptional cases, possible for lower-class groups to capitalize on networks to access the international labour market. The crucial point is that these migrants have to resort to more hazardous step-by-step pathways and hardly ever succeed in overcoming irregular statuses. They are therefore exposed to higher risks to suffer human rights abuses – in transit and in the destination countries.

It is furthermore important to bear in mind that also highly skilled migrants have to navigate the geopolitics of return and that their ideas of development do not always fit what might be defined as Ghana’s national economic interests in political discourses. While skills acquired abroad appeared to be helpful for reintegration, the importance of being able to resort to (pre-)existing social networks—and to combine both new skills and old networks—according to our observations, played an even bigger role in this regard. Moreover, skills obtained abroad were often not compatible with locally required skills and did not contribute to the country’s development as foreseen in AVRR policies: Our results show that highly skilled Ghanaians that were returning from the Global North had more difficulties to adapt to the everyday experience of corruption and favouritism than to develop ideas and businesses.

Differentiating the ways in which migrants navigate governance regimes in their everyday life from a bottom-up perspective also underlines the relevance to look beyond political constraints and individual agency into the specific role of the communities involved: Beyond the characteristics of the individual migrant it is the educational background and social class of the remittance-receivers and the way both interrelate in managing transfers that is decisive: A feature common to migrants regardless of their different levels of skills concerns the sustainability of their remittances in building up reserves for their eventual return. In many cases where investments into real estate or businesses were remotely controlled, they evaporated into thin air once the sender came back. The way in which this affected the reintegration process on the social, economic or political level was yet again dependant on the respective characteristics and the social fabric of the receiving communities.

This, in turn, finally illustrates the multidimensional impacts and the crucial role of the lack of legal pathways for transnational movements—such as trips home—in regard to individual, community and geopolitical aspects. Also the counter-example—those with a legal status abroad—proves the relevance of this matter. The common characteristic of the few returnees that we encountered who have managed to build and maintain houses and businesses in Ghana, was their ability to engage in cyclical return: these persons were able to uphold a stable amount of income from abroad, to personally check their investments in Ghana and to commute back and forth to their respective multiple places of residencies.^12^ Just as for the analytical challenges to define the sustainability of return and reintegration programs within a bipolar model – namely migration versus return – the solution might lie in reconceptualizing this normative and unrealistic model, replacing it with a more descriptive model of circular migration and transnational networks.

## Footnotes

1 https://www.giz.de/en/downloads/giz2019-en-programm-migration-global.pdf

2 https://www.giz.de/en/worldwide/62318.html

3 It is important to highlight that this framing is the prevailing political discourse from the traditional South to North migration perspective. This, however, does not reflect a more nuanced reality in which people also move along the axis from the Global North to the Global South. Destination countries are therefore not only found in the Global North and countries of origin not necessarily located in the Global South.

4 Recent example: “Most Gambians have accused the government of abdicating its responsibilities. ‘This government is not serious about the plight of Gambians both at home and abroad’…" (Takambou 2021).

5 Ever since the transnational turn in migration studies, migration is not regarded as a one-way movement between a point of origin and point of destination anymore. Attention shifted to the ongoing interconnections of migration processes (Vertovec, 2009). It has different meanings and resonances that depend on place, history and circumstances (Rabaka, 2020).

6 This study is part of a larger 3-year long research project, where six researchers and over 10 assistants in the field, investigate the living conditions and livelihood strategies of voluntary returnees (with and without formal assistance) to different countries across West Africa, the Western Balkans and the Middle East. The title of the project is ‘Trajectories of reintegration: the impacts of displacement, migration and return on social change’, and it is funded by the German Federal Ministry for Economic Cooperation and Development (BMZ). The study takes into account the socio-political contexts in countries of origin, transit and destination, the demographic, social, economic and legal characteristics, as well as different framework conditions in the reintegration context.

7 Whereas the general number of migrants heading to Libya slowed down after the war broke out in 2011, male Ghanaian migration from specific regions of the country such as Brong Ahafo to Libya continues to be remarkably high (Darkwah, et al., 2019).

8 Pseudonyms have been used to ensure the respondents’ anonymity.

9 To date, there is little consensus among policy makers, academics and practitioners on how to best define and operationalize migrants’ reintegration (Bilgili & Fransen, 2019). Return migration nevertheless differs from other forms of migration in the way it changes the relationship between the migrant, the place of origin and the people who live there. This relationship determines the potential benefits or pitfalls of return for all parties involved and influences the likelihood of future mobility (Collyer, 2018).

10 Note that at the time being this amount was approximately 20 times the per capita GDP.

11 Collyer (2018) points out that many individuals returning under AVRR schemes go back to situations of generalized violence or war. This hinders access to sustained research. Even in peaceful contexts where access is possible, research faces major issues of identification of returnees.

12 The positive impact on development and family sustenance of such cyclical return is only possible with a legal migration status, which allows people to circulate. Yet, based on the conditions of AVRR programs, for instance, circulation is not the goal of the states in the Global North, which directly questions their real interest in development.
